# Novel concepts for preparation of reference materials as whole water samples for priority substances at nanogram-per-liter level using model suspended particulate matter and humic acids

**DOI:** 10.1007/s00216-014-8349-8

**Published:** 2014-12-07

**Authors:** Saioa Elordui-Zapatarietxe, Ina Fettig, Rosemarie Philipp, Fanny Gantois, Béatrice Lalère, Claudia Swart, Panayot Petrov, Heidi Goenaga-Infante, Guido Vanermen, Gerard Boom, Håkan Emteborg

**Affiliations:** 1 European Commission, Joint Research Centre (JRC), Institute for Reference Materials and Measurements (IRMM), Retieseweg 111, 2440 Geel, Belgium; 2 Bundesanstalt für Materialforschung und -prüfung (BAM), Richard-Willstätter-Str. 11, 12489 Berlin, Germany; 3 Laboratoire National de Métrologie et d’Essais (LNE), 5, Avenue Albert Bartholome, 75015 Paris, France; 4 Physikalisch-Technische Bundesanstalt (PTB), Bundesallee 100, 38116 Braunschweig, Germany; 5 LGC Limited, Queens Road, Teddington, Middlesex, TW11 0LY UK; 6 Flemish Institute for Technological Research (VITO NV), Boeretang 200, 2400 Mol, Belgium; 7 TNO, Princetonlaan 6, 3584 CB Utrecht, The Netherlands

**Keywords:** Slurry, PBDE, PAH, TBT, Reference materials, Suspended particulate matter, Whole water, Water Framework Directive

## Abstract

One of the unresolved issues of the European Water Framework Directive is the unavailability of realistic water reference materials for the organic priority pollutants at low nanogram-per-liter concentrations. In the present study, three different types of ready-to-use water test materials were developed for polycyclic aromatic hydrocarbons (PAHs), polybrominated diphenyl ethers (PBDEs) and tributyltin (TBT) at nanogram-per-liter levels. The first type simulated the dissolved phase in the water and comprised of a solution of humic acids (HA) at 5 mg L^−1^ dissolved organic carbon (DOC) and a spike of the target compounds. The second type of water sample incorporated the particulate phase in water. To this end, model suspended particulate matter (SPM) with a realistic particle size was produced by jet milling soil and sediments containing known amounts of PAHs, PBDEs and TBT and added as slurry to mineral water. The most complex test materials mimicked “whole water” consequently containing both phases, the model SPM and the HA solution with the target analytes strongly bound to the SPM. In this paper, the development of concepts, processing of the starting materials, characterisation of the HA and model SPMs as well as results for homogeneity and stability testing of the ready-to-use test materials are described in detail.

**Graphical Abstract:**
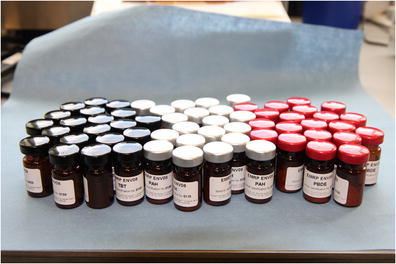
Vials containing 0.5 g of model SPM, black caps for TBT, silver caps for PAH and red caps for PBDEs, respectively.

Vials containing 0.5 g of model SPM, black caps for TBT, silver caps for PAH and red caps for PBDEs, respectively.

**Graphical Abstract:**
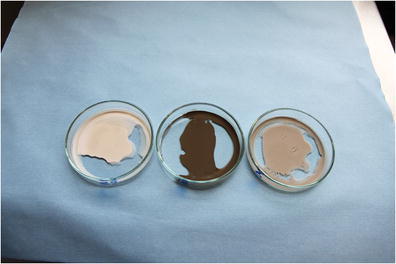
Petri dishes with dried model SPMs; to the left 95.7 ± 0.9 mg of SPM containing PBDEs; in the middle 95.8 ± 0.7 mg of SPM containing TBT and to the right 93.7 mg ± 0.7 mg of SPM containing PAHs

Petri dishes with dried model SPMs; to the left 95.7 ± 0.9 mg of SPM containing PBDEs; in the middle 95.8 ± 0.7 mg of SPM containing TBT and to the right 93.7 mg ± 0.7 mg of SPM containing PAHs

## Introduction

The Water Framework Directive (WFD) 2000/60/EC [1] and its daughter directives are some of the most powerful regulatory tools the European Union currently has to protect the quality of the inland aquatic environment. They are based on the classification of the quality of all European water bodies and application of corrective measures necessary to reach a “good chemical and ecological status” of these water bodies. To this end, Member States must monitor the ecological and chemical status to assess their natural waters. Regarding the most toxic chemical compounds, Directive 2008/105/EC lists Environmental Quality Standards (EQS) [2] for 45 priority substances and their maximum levels. Due to their potentially hazardous nature and widespread occurrence, polycyclic aromatic hydrocarbons (PAHs), polybrominated diphenylethers (PBDEs) and tributyltin (TBT) are among the priority substances.

An additional requirement of the WFD is that it concerns whole, non-filtered waters. The main reason being that suspended particulate matter (SPM) plays a key role in the transport and fate of organic pollutants in the aquatic environment. In fact, the classification of the filtered liquid phase (so-called dissolved) and the SPM phase is operationally defined and not a ‘real’ separation [3]. In natural waters, SPM and colloids represent a dimensional continuum. The “dissolved phase” contains also colloids, mainly humic and fulvic acids and natural macromolecules with a high binding capacity of certain contaminants [4]. PAHs, PBDEs and TBT strongly adsorb to SPM and humic acids (HA) due to their hydrophobic character and can be found in both phases [5, 6]. The partitioning of priority pollutants between the two water compartments depends on several factors including the origin, physico-chemical characteristics of the colloids/SPM, environmental conditions, the hydrological regime and the hydrophobicity of the compounds [5]. It may vary geographically and seasonally [7] and consequently it is important to measure both phases to avoid underestimation of contaminant concentrations [3].

High extraction yields of these hydrophobic organic compounds from non-filtered waters are very difficult to achieve, especially at EQS levels. To date, no validated methods for most of the organic priority substances exist that can be applied to water samples containing substantial amounts of SPM, i.e. up to several hundreds of milligrams per liter [8].

A daughter directive of the WFD (2009/90/EC on technical specifications for chemical analysis and monitoring of water status) [9] requires the use of (certified) reference materials, (C)RMs, if available to ensure the quality of measured data. Their application allows full validation of methods to analyse the priority substances in “whole water” and the establishment of proficiency testing (PT) schemes [10, 11] to ensure that the results are correct, precise and comparable. Unfortunately, such reference materials are not yet available for PAHs, PBDEs and TBT in natural waters. The applicability of developed methods can therefore not be tested on well-characterised reference materials [12].

There have been several attempts to prepare water reference materials with different degrees of complexity for some of the studied compounds. To date, the most widely used approach is reconstitution where participating laboratories prepare the final water sample by spiking a solution of compounds of interest present in a water-miscible solvent into a separately supplied water sample [13–15]. Recently sediment CRMs containing the compounds of interest were added to water, often distilled, as a source of particulates [8]. These sediments add complexity to the water samples but particle sizes are much too large in comparison with particle sizes typically found for SPM. In addition, none of the natural colloids are present. In conclusion, presently, there are no ready-to-use RMs or CRMs simulating whole water available for the organic compounds listed in the WFD.

There are many difficulties to overcome when trying to produce ready-to-use water matrix certified reference materials for hydrophobic organic compounds at low concentrations. The proper preservation of the samples to avoid biological activity, matrix degradation processes and the partitioning of these compounds are still unresolved challenges which result in stability and homogeneity issues [14, 16, 17].

As part of the European Metrology Research Programme, the EMRP-ENV08 project comprised of a feasibility study for the preparation of reference materials for PAHs, PBDEs and TBT in natural waters. The main goal of this study was to develop concepts for the preparation of ready-to-use water “test materials” at the nanogram-per-liter level. In order for the test materials developed in this project to have a meaningful use, they must fulfill the requirements of reference materials [18]. It means that they have to be sufficiently homogeneous and stable for their intended use.

Three types of test materials with different complexities were prepared. The first two water materials contained the dissolved and particulate phases separately and the most complex material contained both phases, hence simulating “whole water”. In this project, the particulate matter comprised of finely milled soil and sediments, hereafter called model SPM. The sample types were subjected to short-term stability (STS), long-term stability (LTS) and homogeneity studies to select the most appropriate concepts for the subsequent steps in the project that resulted in an inter-laboratory comparison.

## Materials and methods

### Cleaning of glass bottles

The bottles were selected after an extensive study carried out to test the suitability of different container materials and container capacities for water test material preparations (Elordui et al. [19]). Amber glass bottles of 1 L capacity (VWR, Leuven, BE) were first cleaned in a lab-grade dishwasher using alkali soap and then manually shaken with a 0.2 % Triton-X 100 solution (Sigma Aldrich, Diegem, BE). Then they were rinsed with Type-1 water (18.2 MΩ cm, 0.053 μS cm^−1^, maximum of 50 μg L^−1^ of total organic carbon and <3 μg mL^−1^ silica at 25 °C according to ASTM D1193-06) [20] and left to dry in a clean cell (Terra Universal, Fullerton, USA) to avoid contamination. Bottles to be used for test materials containing TBT were subjected to an extra cleaning step. In this case, they were left overnight with 6.7 % HNO_3_ (*m*/*m*), rinsed with Type-1 water and then cleaned with the Triton-X 100 solution. The final cleaning step involved a rinse with Suprasolv-grade hexane (Merck, Darmstadt, DE) by swirling about 30 mL over the inner surface of the bottle. The Teflon-lined screw caps were subjected to the same cleaning as reported above depending on use. Bottles were left to dry in the clean cell before being filled with mineral water.

### Preparation and selection of the water matrix

The most suitable water matrix for the test material preparation was selected according to its similarity to natural waters while taking into account several practical aspects. Type-1 water was ruled out because of its low ionic strength, as it would certainly not be representative of any environmental water sample. Use of tap water was also ruled out because chlorinated residues in some tap waters can degrade certain analytes such as naphthalene and benzo(*a*)pyrene [21, 22]. The use of natural waters directly from a lake or a river would require pre-treatment of the water such as filtration, an exhaustive analysis of blanks and, in case of needing extra water, the repetition of the whole process. Bottled mineral water offered a good compromise considering all the aspects mentioned above. It is easily obtained and constitutes a natural matrix free of significant amount of contaminants.

Around 500 L of non-sparkling SPA Reine mineral water (Spa Water, Spa, BE) was obtained in 1-L glass bottles from a local supplier, poured into a perfluoroalkoxy polymer (PFA)-lined tank (Teblick plastic constructions, Wilrijk, BE) and continuously mixed using a IWAKI FS-30HT2 inert bellow pump (Tokyo, Japan). The 1-L VWR amber glass bottles were filled with 1005 ± 3 mL of mineral water using a custom-made system based on displacement of water from a buffer tank by Ar (Teblick plastic constructions, Wilrijk, BE).

### Processing of humic acid solution

A HA solution was prepared following an existing method [23], using technical grade HAs (Sigma Aldrich, St. Louis, USA). 7.5 g of solid was dissolved in 1 L of Type-1 water in an ultrasonic bath and was centrifuged and pooled in a pre-cleaned plastic drum which was left standing overnight to allow sedimentation of particles. Subsequently, the solution was filtered through a 0.8/0.45 μm Versaflow capsule membrane filters (Pall, Farlington, UK) with the aid of a Watson Marlow 624U peristaltic pump (Falmouth, UK). Despite the relatively large surface area of 1390 cm^2^ only about 1 L of HA solution could be filtered before the filter had to be replaced. Finally, the HA solution was filled in 25 mL amber glass ampoules (Nederlandse Ampullenfabriek, Nijmegen, NL) using a 910S ampouling machine (Rota, Baden Wehr, DE). The dissolved organic carbon (DOC) concentration of the HA stock solution was measured in triplicate using a Skalar Formacs TOC analyser coupled to a LAS-160 Autosampler (Skalar, Breda, NL). Blank measurements with respect to PAHs, PBDEs and TBT were also performed using the methodologies listed in Table 1.

**Table 1:** Summary of the methodologies used for the determination of PAHs, PBDEs and TBT in the different preparations

Sample type	Extraction	Determination
PAHs		
Test materials with HA solution and a spike of PAHs	Liquid–liquid extraction	GC-IDMS
Test materials with SPM containing PAHs	Liquid–liquid extraction	GC-IDMS
Test materials with HA solution and SPM containing PAHs	Liquid–liquid extraction	GC-IDMS
Model SPM	ASE	GC-IDMS
PBDEs		
Test materials with HA solution and a spike of PBDEs	Liquid–liquid extraction	GC-IDMS
Test materials with SPM containing PBDEs	Liquid–liquid extraction	GC-ICP/IDMS
Model SPM	ASE	GC-IDMS
	Soxhlet extraction	GC-ECNI/MS
	Extraction by sonication	GC-HRMS
	MAE	GC-MS/MS
TBT		
Test materials with HA solution and a spike of TBT	Liquid–liquid extraction	GC-ICP/IDMS
Test materials with SPM containing TBT	Liquid–liquid extraction	GC-ICP/IDMS
Test materials with HA solution and SPM containing TBT	Liquid–liquid extraction	GC-ICP/IDMS
Model SPM	MAE	GC-MS/MS
	ASE/sonication	GC-ICP/IDMS

*ASE* accelerated solvent extraction; *MAE* microwave assisted extraction

In order to avoid biological activity, the HA stock solution (868 ± 36 mg DOC L^−1^) was irradiated as described in the section “Irradiation of the test materials”.

### Preparation of spiking solutions

#### PAHs

The 8 PAHs listed in the WFD were selected (Table 2). Neat crystals of naphthalene and benzo(*ghi*)perylene were obtained from Fluka (St Louis, USA), anthracene and indeno(1,2,3-*cd*) were obtained from Dr Ehrenstorfer (Augsburg, DE) and fluoranthene, benzo(*b*)fluoranthene, benzo(*k*)fluoranthene and benzo(*a*)pyrene were purchased from Sigma (St Louis, USA). The water samples simulating the dissolved phase were spiked with a standard solution prepared in acetonitrile containing the native compounds in a concentration range of 25.44 to 763.3 ng g^−1^. The solution was stored in the dark at 4 °C until use.

**Table 2:** Theoretical final concentrations of individual PAHs, PBDEs and TBT in the water test materials without SPM and the solvent used to spike them in the water

Compound(s)	Concentration (ng L^−1^)	Solvent
PAHs		
Naphthalene	1200	Acetonitrile
Anthracene	100	
Fluoranthene	100	
Benzo(*b*)fluoranthene	40	
Benzo(*k*)fluoranthene	40	
benzo(*a*)pyrene	50	
Indeno(1,2,3-*cd*)pyrene	40	
Benzo(*ghi*)perylene	40	
PBDEs		
∑BDE28, BDE47, BDE99,		Methanol
BDE100, BDE153, BDE154	4	
TBT	5	Water^a^

^a^The stock solution and intermediate solution were prepared using acetic acid:methanol 3:1 (*v*/*v*); only the last dilution was made using Type-1 water

#### PBDEs

The six PBDEs listed in the WFD were selected in this study (Table 2). The pure PBDE compounds (Accustandard, New Haven, USA), BDE 28, 47, 99, 100, 153 and 154, were accurately weighed into brown glass bottles and gravimetrically dissolved in methanol (SupraSolv, Merck) to result in approximate concentrations ranging from 6.90 to 7.25 ng g^−1^ per congener. The solution was stored in the dark at 4 °C until use.

#### TBT

Water samples were spiked with a tributyltin chloride (Sigma Aldrich, St Louis, USA) solution in water to a concentration of 4.473 ng g^−1^ (as TBT cation). The stock solution was prepared gravimetrically dissolving the TBTCl in a mixture of acetic acid:methanol 3:1 (*v*/*v*). The last dilution step was made with Type-1 water.

### Preparation of model SPMs

Already existing soil and sediment PT materials, CRMs and candidate CRMs having appropriate target concentrations were selected as starting materials (Table 3). These soil and sediments were milled using a jet mill (Alpine, Augsburg, DE) to obtain very fine powders with a top particle size of about 12.5 μm. A similar process as described in the certification report of ERM-CZ100 [24] was applied.

**Table 3:** Characterisation of the model SPMs based on different number of data sets as given in parenthesis below the different SPM-types in the first column, OM Organic Matter (%), and water content, %. Last column shows estimated mass concentrations of individual compounds in 1-L water samples after preparation without including uncertainty due to stability

Model SPM (number of data sets)	Top particle size, *X*_98_^a^	Origin	Water (%)	OM (%)	Estimated mass fraction in model SPM. PAHs / TBT in (μg g^−1^)^b^, PBDEs in (ng g^−1^)^b^	Estimated mass concentration in ready-to-use water samples (ng L^−1^)^c^
PAHs (3)	9 μm	Industrial soil, BAM PT sample	1.10 ± 0.04	6.0 ± 0.03	N, 1.19 ± 0.19	93 ± 15
					A, 0.53 ± 0.07	42 ± 5
					F, 9.51 ± 0.23	742 ± 18
					BbF, 3.11 ± 0.07	243 ± 5
					BkF, 2.31 ± 0.51	181 ± 40
					BaP, 2.33 ± 0.17	182 ± 13
					I, 2.61 ± 0.10	203 ± 8
					BghiP, 3.06 ± 0.07	238 ± 5
PBDEs (4)	9 μm	Freshwater sediment, BE	0.54 ± 0.02	1.4 ± 0.03	BDE28, 0.17 ± 0.01	0.03 ± 0.00
					BDE47, 13.13 ± 0.32	2.55 ± 0.07
					BDE99, 30.50 ± 0.72	5.93 ± 0.15
					BDE100, 4.53 ± 0.14	0.88 ± 0.03
					BDE153, 6.21 ± 0.34	1.21 ± 0.07
					BDE154, 2.89 ± 0.09	0.56 ± 0.02
TBT (6)	12.5 μm	Freshwater sediment BCR-646	2.80 ± 0.25	33.7 ± 0.4	TBT0.50 ± 0.02	3.71 ± 0.12

^a^Size class *X*_98_ refers to 98 % of the particle cumulative distribution being smaller than the particle size given

^b^Spread is given as standard uncertainty of characterisation estimated from the standard deviation of the data set divided by the square root of *n* (number of data sets given in parenthesis for each model SPM)

^c^Estimated mass concentrations based on the masses given in Table 4 to 1 L of water (spread is standard uncertainty including ubb (between-bottle heterogeneity) as standard deviations taken from Table 4 and uncertainty from the SPM characterisation)

### Measurement of particle distribution in model SPMs before and after jet milling

To check the effectiveness of the milling process, particle size analysis (PSA) of the three different types of model SPMs before and after jet milling was performed. The analysis was carried out in a Helos KR laser diffraction system equipped with a 50 mL cuvette wet dispersing system (Sympatec, Clausthal-Zellerfeld, DE). Emsure 2-propanol (Merck, Darmstadt, DE) was used as dispersant.

### Particle size characterisation by field-flow fractionation of model SPM

Small particles (<450 nm) not assessed by the PSA were characterised using field-flow fractionation (FFF) to obtain more information about the nature of the model SPMs concerning sub-micrometer particles. Individual slurries were prepared using the three different model SPMs and were dispersed in Type-1 water to obtain a final concentration of about 200 μg mL^−1^. The samples were stirred in Teflon-lined amber glass bottles overnight with a glass-coated magnetic stirring bar. Next, they were filtered through 450 nm (Whatman Cat. No 6880-1304 (LOT 115278)) and 200 nm (Whatman Cat No. 6880-1302 (LOT 99874)) syringe filters (Fisher Scientific, Loughborough, UK).

The samples were fractionated using a AF2000 Asymmetrical Flow Field-Flow Fractionation system (Postnova, Landsberg am Lech, DE) with a multi-step power-field decay program. The AF200 system was equipped with 1-kDa PES membrane and a 500 μm spacer. A multi-angle light scattering (21 angles) and an UV detector were connected online for the detection of the particles. Particle multi-angle light scattering (MALS) at 13 scattering angles (between 28° and 124°) was used for particle size measurements. The detector size calibration was checked using 100 nm diameter (product code: Z-PS-POS-825-0,1) and 200 nm diameter (product code: Z-PS-POS-825-0,2) polystyrene S-PS spherical particles from: Postnova Analytics GmbH. The obtained particle size accuracy of the calibrants was within the acceptable range as given by the manufacturer (±15 % of the conceptually true value). UV absorption spectra at 254 nm (main wavelength) and 236 nm (reference) were recorded throughout the fractionation process. The data acquisition rate was 0.5 Hz.

MALS provides information about the geometrical mean spherical radius at a given time and the particle diameters are then calculated. A conversion factor of 1.29 is applied to calculate the actual particle diameter as dictated by light-scattering theory.

### Measurement of water content in model SPMs

The model SPMs for PAHs, PBDEs and TBT were checked with respect to water content using volumetric Karl Fischer titration 758 KFD Titrino (Metrohm, Herisau, CH). Four vials were analysed in duplicate with reagents from Riedel-de Haën, Seelze, DE.

### Measurement of ash content in model SPMs

The organic content in the SPM samples was determined by loss of ignition [4]. About 2 g of each model SPM were weighed in glass microfiber crucibles and fired in a Phoenix CEM ashing microwave furnace (CEM, Matthews, USA) at 750 °C for 40 min. They were left to cool down in a desiccator overnight and then weighed again. All model SPMs were analysed in duplicate.

### Determination of PAHs, PBDEs and TBT in the three different types of test materials

The determination of PAHs, PBDEs and TBT in model SPMs and test materials was carried out by different laboratories using their in-house methodologies. In Table 1, a general summary of each methodology is given.

### Methodology for addition of model SPM to water

The slurry was prepared by suspending the model SPMs in Type-1 water under constant mixing using a glass-coated magnetic stirring bar at 500 rpm. Small volumes of this slurry were pipetted into the bottles pre-filled with mineral water using a transfer vial. The small accurately weighed transfer vial was used to ensure quantitative material transfers. The mineral water supplied in the bottle was used to rinse out the transfer vials and pipet tips for all SPM loadings. The transfer was checked using a balance with a resolution of 0.1 mg. The sampling from the continuously stirred slurry was done by introducing the pipette well under the surface but avoiding the bottom, as recommended in previous studies [25].

### Irradiation of the test materials

Irradiation of the ampoules with HA stock solution and 1-L samples was performed by Synergy Health Ede B.V., Etten-Leur, NL. The test materials containing PAHs and TBT were given a dose from 9.4 to 11.3 KGy. The 25-mL ampoules containing the concentrated HA solution were irradiated with a dose of 15.3–17.8 kGy.

## Results and discussion

### Development and assessment of test materials

**Fig. 1:**
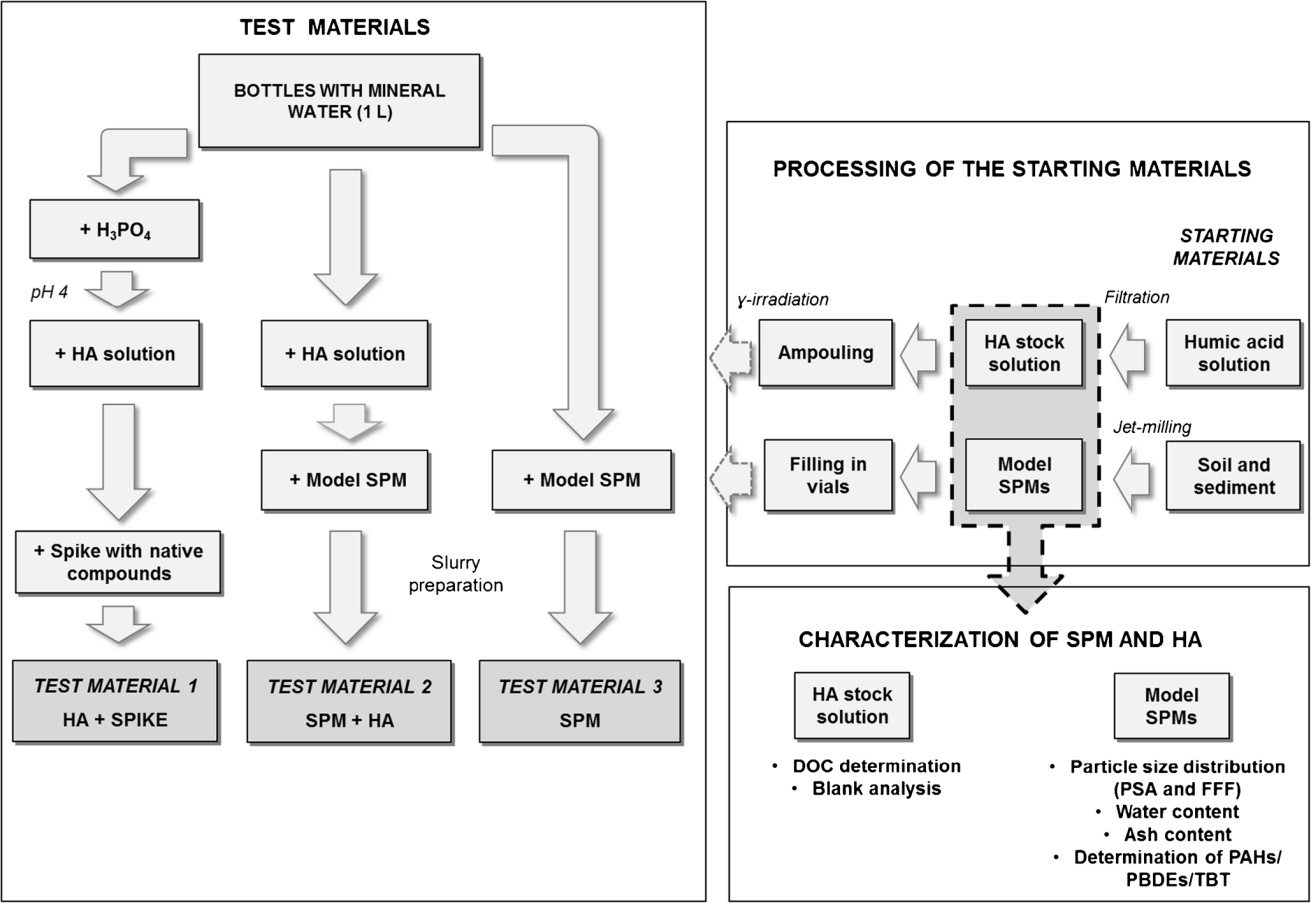
General scheme of the steps followed in the preparation of the three types of water testing materials. On the *top right* the processing of the starting materials is shown; on the *bottom right* the different tests performed on the model SPMs after processing, and on the *left* the different steps of the final test material preparations are displayed

Three types of test materials were produced simulating different water phases as shown in Fig. 1:

- *Dissolved phase*: Containing dissolved HA and a spike with target compounds
- *Particulate phase*: Containing SPM
- *Whole water*: Containing SPM and dissolved HA

Independent materials were prepared for each compound group resulting in eight different test materials: three types for PAHs (HA + spike, SPM, SPM + HA), three types for TBT (HA + spike, SPM, SPM + HA) and two types for PBDEs (HA + spike, SPM).

#### Test materials containing humic acids and a spike

The main challenge for the preparation of test materials representing the dissolved phase was to keep the target compounds in solution. Due to their hydrophobic nature, they tend to adsorb onto container walls [26, 27]. In order to keep the spiked compounds in the dissolved phase and make the matrix as realistic as possible, a solution of commercially available HA mimicking natural colloids was added to mineral water. Not only does the HA act as a ligand for the target compounds [7, 28] but their addition to the water also poses an additional analytical challenge for the determination of the priority pollutants [23].

To avoid the precipitation of HA, mineral water was acidified to pH 4 by adding 1 mL of 2 % H_3_PO_4_ (*v*/*v*) to pre-filled bottles. Next, the HA stock solution was added to a final concentration of about 5 mg L^−1^ DOC, a level found in many natural waters [29, 30]. Finally, 1 mL of a solution containing the target compounds in a water-miscible solvent like methanol or acetonitrile was added (Table 2) and the content of the bottles was thoroughly mixed.

The final concentrations of different compounds in the test materials were a compromise between concentrations close enough to EQS levels and analytical capabilities to be able to reliably measure the compounds in the presence of humic acids.

#### Test materials containing SPM

The materials with SPM alone were prepared adding 1 or 2 mL of slurry of the model SPM to the bottles pre-filled with mineral water to obtain the desired concentrations (Table 4).

**Table 4:** Repeatability of mass of model SPM sampled by pipetting the slurry from different testing material preparations

Model SPM	Preparation	Slurry (mL)	Model SPM (mg)	RSD (%)
PAHs	Model SPM, 7.94 g	1	78.03 ± 0.14	0.18
	Type-1 water, 99.59 g			
PBDEs	Model SPM, 11.99 g	2	194.50 ± 0.85	0.44
	Type-1 water, 119.26 g			
TBT	Model SPM, 0.73 g	1	7.46 ± 0.08	1.1
	Type-1 water, 103.3 g			

Spread is given as ±one standard deviation of the mean

*RSD* relative standard deviation (%); *SPM* suspended particulate matter, *n* = 6 for each SPM

The final SPM concentration in the samples ranged from about 7.46 to 194.5 mg L^−1^ (Table 4), which is within the range found in natural waters [31]. The SPM load for different test materials was dictated by the intended final concentration of target compounds in the water samples and the actual concentrations of PAHs, PBDEs and TBT content in the model SPMs as given in Table 3.

Final concentrations of PAHs, PBDEs and TBT in the test materials were as close as possible to the EQS levels always taking into account the limitations posed by the distribution of individual compounds in the model SPM for PAH and PBDE (Table 3).

#### Test materials containing SPM and HA solution

The most complex material contained both HA and SPM and mimicked “whole water”. It was prepared by adding HA stock solution to the water until reaching 5 mg L^−1^ DOC and the same amount of SPM as in samples without added HA. In this case, the acidification of the water prior to the addition of HA was avoided so as not to result in changes in the phase distribution of the target compounds due to drastic pH changes [32]. In both materials containing SPM, no extra spike of the target compound was added, since the contaminants are already present and strongly bound to the SPM.

### Production of model SPM

The main challenges in the preparation of test materials with SPM were to obtain appropriate SPMs and to device a reproducible method for their addition to the 1-L water samples. Collection of natural SPM for this purpose was not considered for several reasons: This being laborious to sample, large amounts were needed and the collected SPM had to be thoroughly homogenised and characterised for the target compounds. In case more SPM would be required at some point during the project, all these steps also would have to be repeated.

As an alternative to a natural SPM, the production of model SPMs was chosen using soil and sediment starting materials with known concentrations of the compounds of interest. The advantages of this approach were that the starting materials could be readily obtained, were already well-characterised and sufficient amounts of model SPMs could be prepared. The major challenge was reduction of particle size to be close enough to top particle sizes encountered for natural SPMs [4].

#### Characterisation of the model SPM

**Fig. 2:**
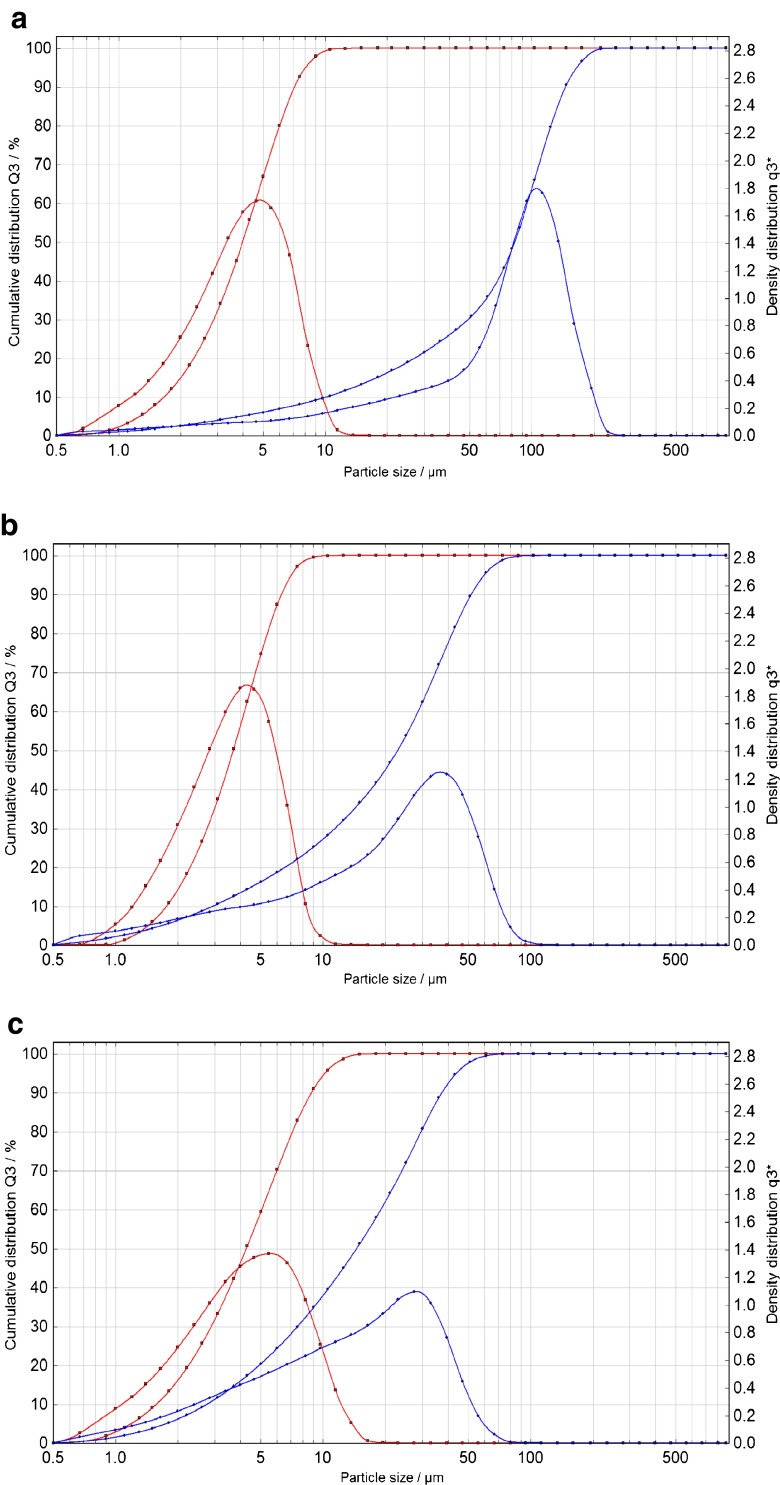
Particle size analysis (PSA) in the starting soil and sediment materials (*blue*) and the corresponding processed model SPMs (*red*) for PAHs (**a**), PBDEs (**b**) and TBT (**c**)

In natural waters, the particle size of SPM ranges from 0.003 to >25 μm as reported by Ran et al. [4]. The laser diffraction technique showed the presence of particles in the range from 0.5 to 12.5 μm (Fig. 2) although smaller particles are also present as measured by FFF-MALS/UV. Obviously water systems have different SPM loads and characteristics but for the purpose of this study the chosen approach was deemed to be appropriate. Similarly, it is recognised that most of the model SPM would precipitate in a static system (i.e. a glass bottle with water standing still). Therefore, it is essential that the bottles were vigorously shaken before analysis for quantitative recoveries. No subsampling is possible since all water and added SPM must be analysed per bottle.

Several properties of the model SPM were measured to better understand their behaviour when preparing slurries (Table 3). The TBT SPM is rich in organic material (33 % of organic matter (OM) content) while the PBDE SPM is poor in organic matter (1.4 % of OM). This variability can be explained due to their contamination history or influence of effluents of anthropogenic origin [33]. It was also observed that in the prepared model SPMs, the water content was related with the organic matter amount, the higher the OM concentration the higher the water content. A similar correlation has previously been observed for these two parameters in previous studies [34].

Substantial efforts were also made to carefully characterise all model SPMs with respect to the target analytes after jet milling (Table 3). For each model SPM, several individual data sets were obtained which agreed well with the certified value and/or between data sets.

**Fig. 3:**
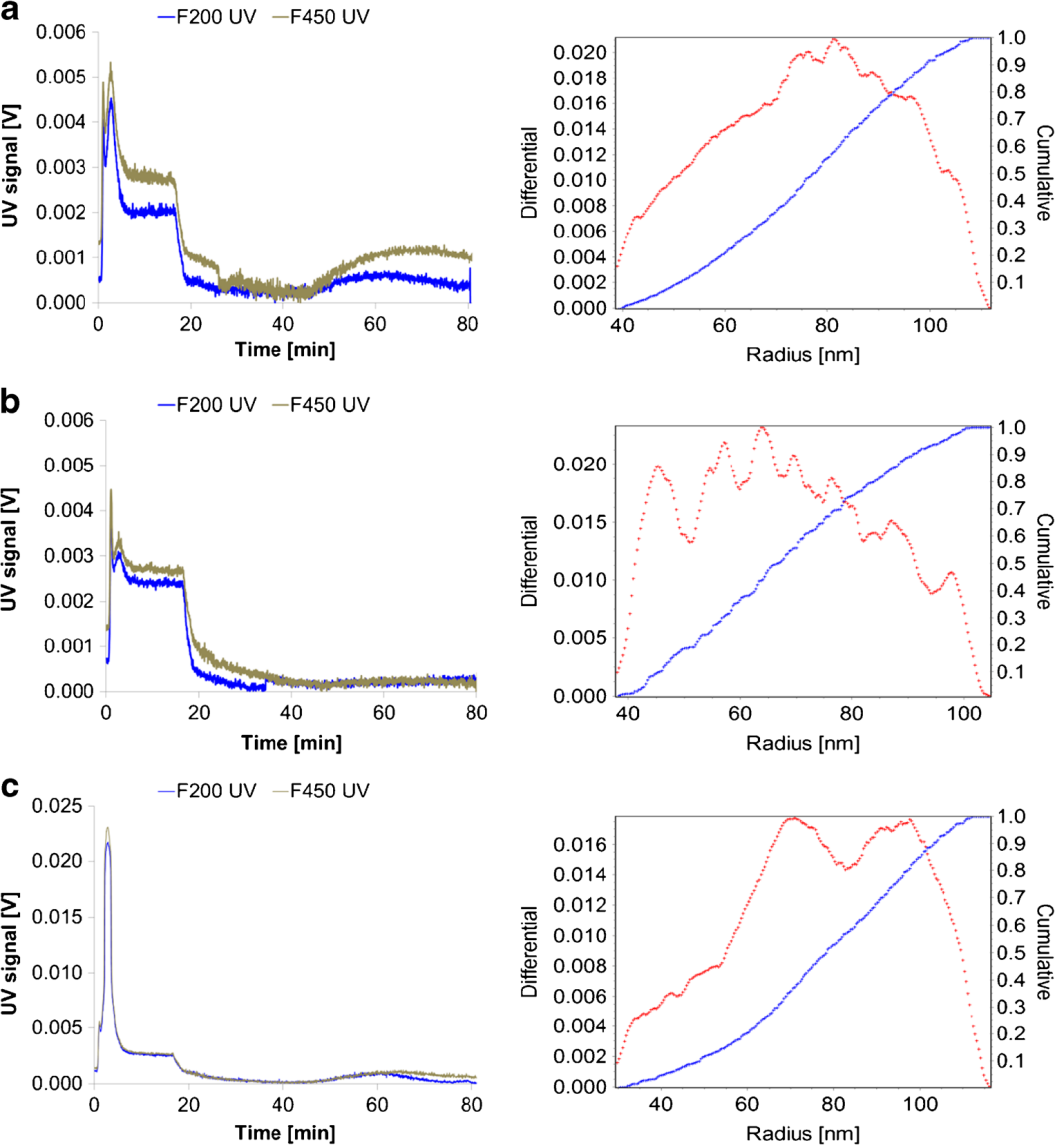
Particle size distribution of the SPM containing PAHs (**a**), PBDEs (**b**) and TBT (**c**) by field-flow fractionation (FFF). On the *left*, overlaid UV fractograms (graphs of a detection signal vs. time) of 200 nm and 450 nm PBDE SPM filtrates are shown. On the *right*, the geometrical mean spherical radius is displayed of a 450-nm filtrate as determined by MALS detection

Particles smaller than 0.45 μm and their size distribution as measured by FFF are displayed in Fig. 3. Naturally occurring particles smaller than 0.45 μm are not clearly defined spherical particles but rather a complex mixture of particles of different shapes and possibly agglomerates of very small particles. The FFF results should be considered in this context and that a spherical calibration and fit has been used to characterise this fraction by MALS. In Fig. 3a, the PAH SPM is displayed where the particles ranged from about 100 to 300 nm. Small organic particles below 20 nm as detected by UV can be observed but in rather low amounts. In Fig. 3b, the PBDE SPM is displayed which has particles with diameter from about 100 to 270 nm with a polymodal distribution and is lacking organic particles below 20 nm. In Fig. 3c, the TBT SPM is shown where most of the particle sizes ranged from approximately 100 nm to 300 nm which are assumed to be agglomerates of smaller particles. There is a high amount of small-sized particles below 20 nm with a high OM content (represented by the early UV peak in Fig. 3c) which correlates well with the high OM content reported in Table 3.

#### Slurry addition

The method of addition of SPMs to the 1-L water samples had to be highly reproducible, quantitative, easy to perform and not introduce significant between-unit heterogeneity between the testing samples. Therefore, the methodology for the test material preparations including SPM was based on repeatable pipetting of aliquots of a continuously mixed slurry to pre-filled water bottles.

Theory and practice of slurry sampling has been extensively studied since finely milled powders have already been introduced directly in graphite furnaces as slurries with subsequent detection using atomic absorption spectrometry (AAS) [35]. The main uncertainties related to this type of sampling come from the particle size and particle distribution in the slurry mixture. It has been empirically proven that smaller particle sizes and narrower distribution ranges reduce the inhomogeneity of the samples [36]. Accurate measurements have been obtained using particles up to 25 μm [35, 37] which is more than twice the size in comparison to the top particle size of the model SPMs used in this study. Obviously trace elements were measured using ETAAS but the basic theory is the same.

There are several advantages of choosing slurry spiking over direct dry mass addition: the volumes of aliquots (1 and 2 mL) are much easier to handle than directly adding portions of 7.5 mg of fine powder, i.e. in the case of TBT. Moreover, the repeatability of the added aliquot amount is much higher and the heterogeneity between each aliquot is much smaller, since the slurry comprises of several pooled units of model SPM. The individual particles, on which the analytes are attached, are in constant motion in the stirred slurry, and therefore also continuously homogenised during stirring and sampling.

The increased homogeneity of the analyte concentration in slurries was also assessed by measurements. Dry mass addition and slurry sampling were compared, determining the concentration of PAHs in the model SPM (Table 5). Lower relative standard deviations were obtained for 5 replicate measurements of 20 mg SPM added as slurry in comparison to 20 replicate measurements of 200 mg of dry powder. No tests were done for PBDEs for dry powder sampling, but the results obtained preparing a slurry at 20 mg L^−1^ resulted in RSDs ranging from 3.5 to 8.7 % in the picogram-per-liter range for some of the tested PBDE congeners. These results confirm that the processed SPMs are homogeneous, and that slurry addition is an extremely reproducible technique for the addition of SPM to test materials.

**Table 5:** Repeatability of the PAHs and PBDEs in the model SPM analysed directly or analysed after slurry addition. Relative standard deviations (RSD) were calculated for 20 replicates (PAHs, 200 mg, dry mass), 5 replicates (PAHs, 20 mg, slurry addition) and 4 replicates (PBDEs, 20 mg, slurry addition)

	RSD (%) Dry powder analysis, 200 mg	RSD (%) Slurry analysis, 20 mg
PAHs		
Naphthalene	3.1	2.2
Anthracene	4.8	2.6
Fluoranthene	4.1	2.0
Benzo(*b*)fluoranthene	4.0	4.8
Benzo(*k*)fluoranthene	13.2	2.2
benzo(*a*)pyrene	4.2	1.6
Indeno(1,2,3-*cd*)pyrene	5.7	2.4
Benzo(*ghi*)perylene	4.8	2.2
PBDEs		
BDE28	–	8.7
BDE47	–	7.8
BDE99	–	3.7
BDE100	–	3.5
BDE153	–	4.7
BDE154	–	4.6

To be able to produce several dozens of test samples from the same slurry, the release of the target compounds from the SPM to the Type-1 water was also studied. In case of a significant desorption into the liquid phase, the target analytes might be subjected to losses such as evaporation and/or adsorption to the container walls which could introduce a trend in the added amounts of target analytes. Therefore, 1 g of the model SPM was added to 1 L of Type-1 water and then the slurries were mixed for 10 min and 1 h, respectively (*n* = 2 for each time point). At these two time intervals, the preparations were filtered through a 0.8/0.45 μm filter and the target compounds were measured in the filtrates. Only in the case of naphthalene, the lightest, most polar PAH and therefore most soluble compound, was detected in the filtrate at a moderate concentration with a significant difference between 10 min and 1 h of stirring, respectively. For the remaining PAHs, PBDEs and TBT, less than 1 % of the compounds were found in the filtrates. This finding agrees with similar experiments [38] where desorption of PAHs from river sediment CRM was found to be of the same magnitude (<1 %). Consequently, the amount of the target analytes released to the water from the model SPM during the sample preparation step was insignificant, with the exception of naphthalene. No major problem (induced heterogeneity or trend) should therefore arise when preparing numerous test samples from the same slurry over a period of 1 h.

#### Control of SPM load and between-bottle heterogeneity

During the preparation of SPM and SPM + HA type of materials, the amount of SPM added to the samples was always controlled by placing 1 or 2 mL of the slurry in pre-weighed petri dishes (*n* = 6). Two petri dishes were filled before adding the slurry to the water samples, two in the middle of the preparation sequence and two at the very end. The petri dishes were left to air dry in a clean bench and the dry mass of the loaded SPM was calculated. In this manner, it was possible to assess if there was a difference in the SPM density over time in the constantly mixed slurry. The relative standard deviation (RSD) of the aliquots of SPM taken from the slurry ranged from between 0.18 to 1.1 % for all the prepared materials from the beginning to the end of the sequence as shown in Table 4. Therefore, it was concluded that there were no significant variations in the sampling of SPM from the slurries during the material preparation process. In addition, no trend in the sampled amount could be observed either. Because of the low variability of the SPM sampling, it was possible to prepare up to 50 samples with low between-bottle heterogeneity.

### Stability of the test samples

Initially, the effect of gamma-irradiation and storage temperature on PAHs and TBT test materials was studied. Replicates of the three test material types (HA + spike, SPM and HA + SPM) were prepared individually for PAHs and TBT. After being irradiated, their stability was studied at 4, 18 and 60 °C for a period of 7 weeks.

**Fig. 4:**
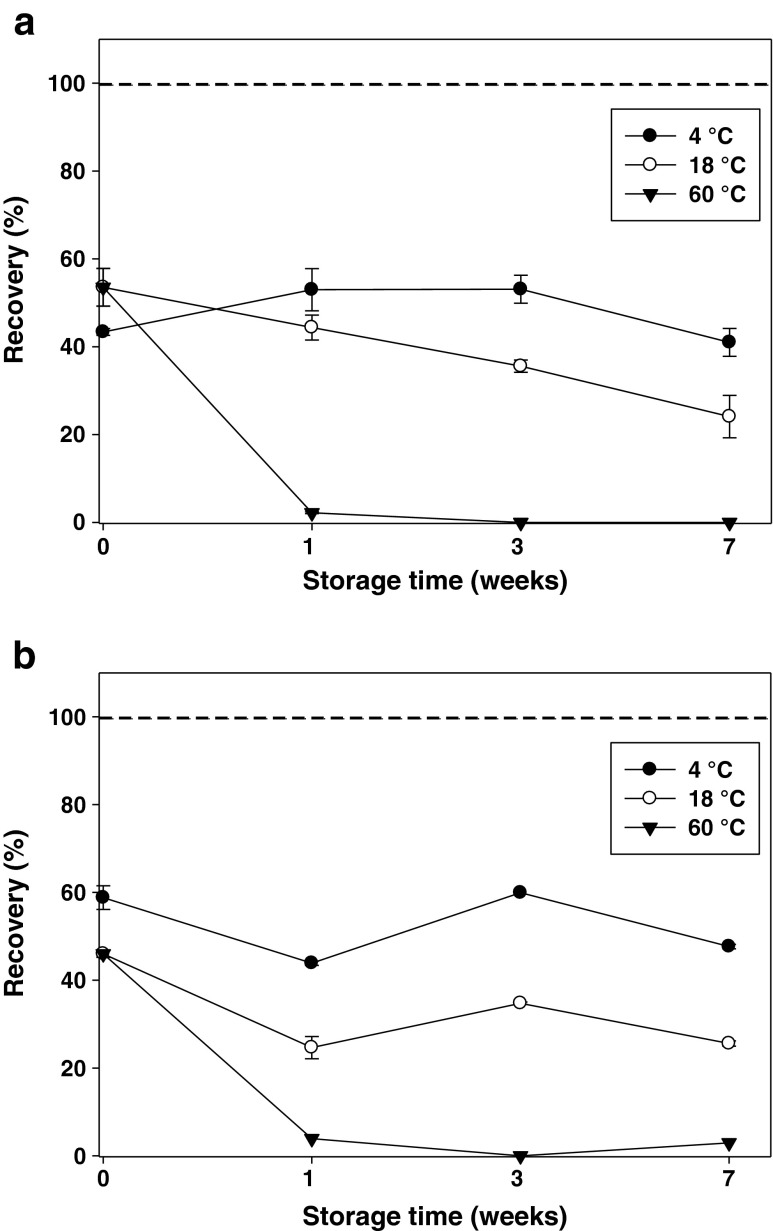
Effect of irradiation on TBT test materials containing SPM (**a**) and SPM + HA (**b**) and stored at different temperatures. The *dotted line* (100 %) corresponds to the concentration in non-irradiated reference samples (*t* = 0 weeks). *Error bars* represent ±one standard deviation of the measurement mean

The initial expectations that a relatively low dose of γ-irradiation would stabilise the samples and the analytes were unfortunately not confirmed since losses of analyte were incurred by irradiation. The degree, to which the compounds are affected by γ-irradiation, depends greatly on their distribution between the different phases in the water. The irradiation affected TBT both in the dissolved phase and in the SPM phase but while TBT disappeared completely in the dissolved phase (below detection limit), quantifiable amounts of TBT remained intact on the model SPM (Fig. 4). For these samples, about 50 % of the TBT remained after gamma-irradiation as can be observed comparing with the irradiated reference samples (at *t* = 0 weeks) to the reference samples without irradiation (represented as the 100 % line). The shielding effect coming from the SPM has been observed previously. A similar study [39] showed that only 38 % of the TBT spiked into soils was degraded while up to 90 % loss occurred in a methanol solution upon irradiation (dose of 25 kGy). Similar results were obtained for PAHs.

Despite the results discussed above, the suitability of γ-irradiation for the preservation of ready-to-use water testing materials should not be discarded for future experiments, especially when the compounds are bound to SPM [39]. Nevertheless, detailed experiments to adjust the optimum irradiation dose to obtain an efficient sterilisation of the water while minimizing the effect on the target compounds should be performed. Likewise, all bottles should be irradiated in the same way, which is challenging due to shielding effects in the pallet irradiator.

For PAHs and TBT, the temperature also plays a key role for their stability during the testing period. TBT almost completely disappeared after 1 week of exposure to 60 °C. Similarly, losses up to 87 % were observed for some of the PAHs after 7 weeks under the same conditions. Testing of the stability of the materials at 60 °C is applied to investigate effects of harsh but not unrealistic transport conditions for CRMs during the so-called short-term stability studies, STS. Clearly high temperatures must be avoided for the prepared test materials during shipment.

**Fig. 5:**
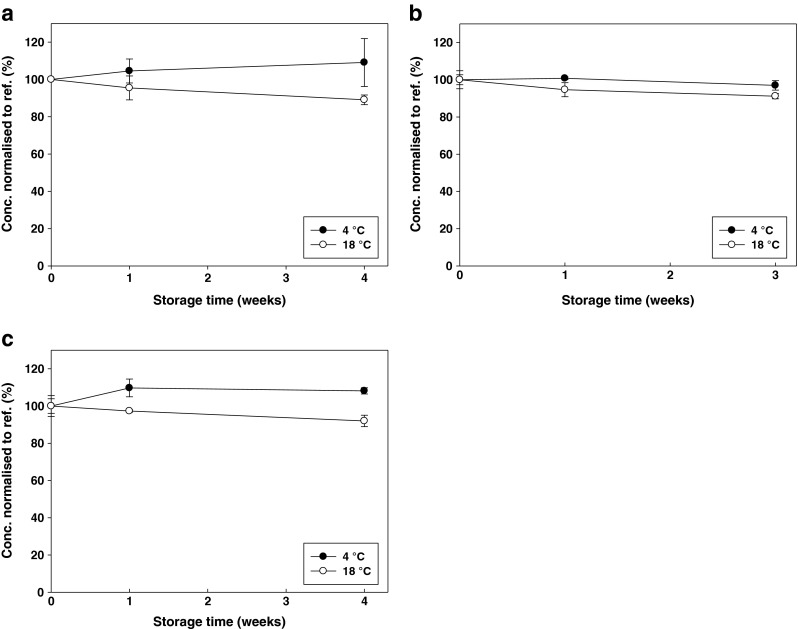
Example for the short term stability of the target analytes in the PAH (**a**), PBDE (**b**) and TBT (**c**) test materials containing SPMs over 4 weeks. Benzo(*ghi*)perylene and BDE100 are shown as representative of PAHs and PBDEs, respectively. *Error bars* represent ±one standard deviation of the measurement mean

The studied compounds are generally stable for 4 weeks at 4 °C as confirmed during STS studies performed with PAHs/PBDEs/TBT on non-irradiated samples as shown in Fig. 5 (not all results shown).

It was decided to keep all the samples at 4 °C in the dark from preparation until analysis. Moreover, the planned inter-laboratory comparison as outlined in the introduction relied on express overnight courier shipments to laboratories in Europe (intercomparison results to be reported elsewhere).

## Conclusions

Three types of ready-to-use water test materials were successfully developed for PAHs, PBDEs and TBT at nanogram-per-liter levels. The material containing dissolved humic acids and model SPM is a step further in the production of test materials mimicking whole water as stipulated in the European Union Water Framework Directive.

Jet milling of soils and sediments with known or certified pollutant concentrations is an attractive alternative for the production of model SPMs for the subsequent preparation of water test materials. The small particle size obtained offers a realistic approach to simulate the particulate phase in the water in contrast to the addition of coarser CRMs to water samples.

Aliquot sampling from continuously stirred slurries proved to be a very reproducible and reliable method to add model SPMs to pre-filled water bottles with the prospect of automation. In this way, it was possible to produce ready-to-use testing materials with low between-unit heterogeneity as proven by actual measurements at nanogram-per-liter level. It also allows the preparation of a larger number of water samples in one campaign and is therefore a suitable method for producing water samples necessary for PT schemes or potential candidate CRMs.

Long-term stability of water materials is still an unresolved issue. At tested conditions, gamma-irradiation was found to be unsuitable for conservation of prepared samples. Sufficient proof of stability was gathered for samples kept at 4 °C in the dark for at least 4 weeks. Future developments may require supplying SPM and the water matrix separately and distribute as a kit or finding γ-irradiation conditions that do not cause major analyte break-down in ready-to-use materials. Nonetheless, the findings presented here constitute a significant improvement with respect to reference sample preparation of more realistic water samples.
